# mRNA-lncRNA gene expression signature in HPV-associated neoplasia and cervical cancer

**DOI:** 10.18699/vjgb-24-39

**Published:** 2024-06

**Authors:** E.D. Kulaeva, E.S. Muzlaeva, E.V. Mashkina

**Affiliations:** Southern Federal University, Rostov-on-Don, Russia; Southern Federal University, Rostov-on-Don, Russia; Southern Federal University, Rostov-on-Don, Russia

**Keywords:** human papillomavirus, neoplasia, cervical cancer, transcriptome analysis, lncRNA, CDKN2A, CCL20, вирус папилломы человека, неоплазия, рак шейки матки, транскриптомный анализ, lncRNA, CDKN2A, CCL20

## Abstract

Cervical cancer is one of the most frequent cancers in women and is associated with human papillomavirus (HPV) in 70 % of cases. Cervical cancer occurs because of progression of low-differentiated cervical intraepithelial neoplasia through grade 2 and 3 lesions. Along with the protein-coding genes, long noncoding RNAs (lncRNAs) play an important role in the development of malignant cell transformation. Although human papillomavirus is widespread, there is currently no well-characterized transcriptomic signature to predict whether this tumor will develop in the presence of HPV-associated neoplastic changes in the cervical epithelium. Changes in gene activity in tumors reflect the biological diversity of cellular phenotype and physiological functions and can be an important diagnostic marker. We performed comparative transcriptome analysis using open RNA sequencing data to assess differentially expressed genes between normal tissue, neoplastic epithelium, and cervical cancer. Raw data were preprocessed using the Galaxy platform. Batch effect correction, identification of differentially expressed genes, and gene set enrichment analysis (GSEA) were performed using R programming language packages. Subcellular localization of lncRNA was analyzed using Locate-R and iLoc-LncRNA 2.0 web services. 1,572 differentially expressed genes (DEGs) were recorded in the “cancer vs. control” comparison, and 1,260 DEGs were recorded in the “cancer vs. neoplasia” comparison. Only two genes were observed to be differentially expressed in the “neoplasia vs. control” comparison. The search for common genes among the most strongly differentially expressed genes among all comparison groups resulted in the identification of an expression signature consisting of the CCL20, CDKN2A, CTCFL, piR-55219, TRH, SLC27A6 and EPHA5 genes. The transcription level of the CCL20 and CDKN2A genes becomes increased at the stage of neoplastic epithelial changes and stays so in cervical cancer. Validation on an independent microarray dataset showed that the differential expression patterns of the CDKN2A and SLC27A6 genes were conserved in the respective gene expression comparisons between groups.

## Abstract (ru)

Рак шейки матки является одним из наиболее частых онкологических заболеваний у женщин и в 70 % случаев связан с вирусом папилломы человека (ВПЧ). Рак шейки матки развивается в результате прогрессии цервикальной интраэпителиальной неоплазии через поражения второй и третьей степени. Помимо белок-кодирующих генов, важную роль в развитии злокачественной трансформации клеток играют длинные некодирующие РНК. Хотя вирус папилломы человека широко распространен, в настоящее время нет хорошо охарактеризованных транскриптомных признаков, позволяющих предсказать злокачественную трансформацию клеток эпителия при наличии связанной с ВПЧ неоплазии эпителия шейки матки. Изменения генной активности в опухолях отражают биологическое разнообразие клеточного фенотипа и физиологических функций и могут быть важным диагностическим маркером. Используя открытые данные секвенирования РНК, мы провели сравнительный анализ транскриптома для оценки дифференциально экспрессируемых генов в образцах нормальной ткани, эпителия с диспластическими изменениями и раком шейки матки. Первичные данные были предварительно обработаны с использованием платформы Galaxy. Коррекция пакетного эффекта, идентификация дифференциально экспрессируемых генов и анализ обогащения набора генов выполнены в пакетах языка программирования R. Субклеточная локализация днРНК была проанализирована с помощью веб-сервисов Locate-R и iLoc-LncRNA 2.0. В сравнении «рак vs. контроль» зарегистрировано 1572 дифференциально экспрессируемых гена, в сравнении «рак vs. неоплазия» – 1260. Только два дифференциально экспрессируемых гена выявлено при сравнении контроля и неоплазии. Поиск общих среди наиболее сильно дифференциально экспрессируемых генов во всех группах сравнения привел к выявлению сигнатуры экспрессии, состоящей из генов CCL20, CDKN2A, CTCFL, piR-55219, TRH, SLC27A6 и EPHA5. Повышенный уровень транскрипции генов CCL20 и CDKN2A возникает на стадии неопластических изменений эпителия и сохраняется при раке шейки матки. Валидация на независимом наборе данных микрочипа показала, что паттерны дифференциальной экспрессии генов CDKN2A и SLC27A6 сохраняются в соответствующих сравнениях экспрессии генов между группами

## Introduction

Cervical cancer is the fourth most common cancer in women worldwide after breast cancer, colorectal cancer, and lung cancer. The World Health Organization (WHO) estimates that 604,127 new cases and 341,831 deaths from the disease worldwide were registered in 2020 (Sung et al., 2021; Gebrie, 2022). Cervical cancer occurs as a result of progression of low-differentiated cervical intraepithelial neoplasia (CIN1) through grade 2 and 3 lesions (CIN2 and CIN3). Inflammatory responses are rarely observed in persistent low-grade lesions and are thought to be due to the inflammation-suppressing activity of high-risk HPV oncoproteins (Walch-Ruckheim et al., 2015).

Although HPV is the most significant factor in cervical cancer, the development of cervical cancer is considered multifactorial. Common risk factors for cervical cancer also include smoking, a high number of sexual partners, low social and/or economic status and its consequences, and immune suppression caused by infection such as human immunodeficiency virus (HIV) or the use of immunosuppressants after organ transplantation (Walch-Ruckheim et al., 2015).

According to the International Human Papillomavirus Reference Center data (Eklund et al., 2020), only 12 out of 220 HPV strains have the greatest impact on cancer development (these strains include HPV types 16, 18, 31, 33, 35, 39, 45, 51, 52, 56, 58, and 59). About 70 % of cervical cancer and precancerous lesions of the cervix cases are specifically associated with HPV types 16 and 18 (Okunade, 2020).

Eighty percent of sexually active women become infected with HPV during their lifetime, but the infection persists in only 5–10 % of those initially infected and leads to cervical cancer in only 3 % (Schubert et al., 2023). In the absence of a clearly persistent HPV infection, the risk of developing cervical cancer is extremely low. However, virus persistence may be associated with many factors. Host genetic factors are thought to play an important role in the response to HPV infection and further development of oncology.

Along with the protein-coding genes, long noncoding RNAs (lncRNAs) play an important role in the development of malignant cell transformation. Results of the TCGA project showed that approximately the same number of proteincoding genes and lncRNA genes carried mutations in more than 5,000 different tumor samples. However, at the same time, 60 % of lncRNAs showed tumor type specificity and are superior to protein-coding genes in terms of specificity to the type of cancer (Yan et al., 2015). Consequently, lncRNAs can be a good class of biomarkers for cancer prognosis and early diagnosis.

Both protein-coding and lncRNAs can be analyzed as efficiently as possible by high-throughput RNA sequencing (RNA-seq). Profiling the entire transcriptome can identify genes that are differentially expressed in related tissues. Changes in gene activity in tumors reflect the biological diversity of cellular phenotype and physiological functions and can be an important diagnostic marker (Martin, Wang, 2011; Bao et al., 2019). A significant change in the expression of both protein- coding and non-coding parts of the genome may be a consequence of local chromatin remodeling in the region of the virus integration site, which plays a somewhat spontaneous but often important role in oncogenesis (Karimzadeh et al., 2023).

The aim of this work was to perform bioinformatics analysis of RNA sequencing data from epitheliocytes of women with cervical epithelial neoplasia and cervical cancer based on open data from three different studies (Royse et al., 2014; Hu et al., 2015; Qi et al., 2022).

## Materials and methods

Datasets. The study material was raw RNA sequencing data of cervical epithelial samples from three separate studies analyzing the transcriptome in cervical cancer, neoplasia, and normal tissue. Neoplasia grade data were also available. The main characteristics of the studies used are summarized in Table 1.

**Table 1.**
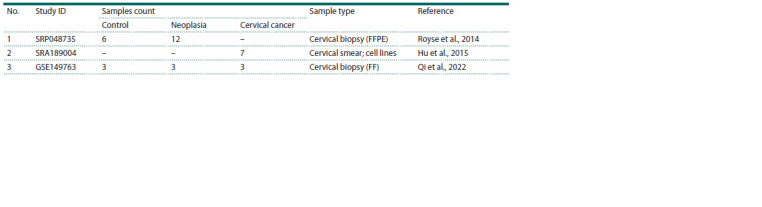
Main characteristics of the used studies

Data preprocessing. Raw RNA-seq data (fastq format) were processed using the Galaxy platform (https://usegalaxy. org/). Read quality was assessed with FastQC, adapter trimming was performed with TrimGalore, transcript alignment and mapping was performed with RNA STAR, and transcript counting was performed with featureCounts, respectively.

Data variability analysis and batch effect correction. Analysis of data variability and assessment of the batch effect (effect of the subsample/sequencing platform rather than biological variability) were performed using principal component analysis (PCA) with the plotPCA function of the DESeq2 v.1.42.0 package for R. Based on the results of the variability analysis, a conclusion was made about the inclusion/exclusion of samples in further analysis.

Differential gene expression analysis was performed using the DESeq2 package in R. Genes were filtered by log2FC >2, log2FC < (–2), and adjusted p-value < 0.05 (as visualized in the R package EnchancedVolcano). Genes encoding mRNAs and lncRNAs were categorized using Ensembl Ids. To identify differentially expressed genes (DEGs) between three biological states (neoplasia vs. control; cancer vs. neoplasia; cancer vs. control), comparisons were performed and the top 10 genes with statistically significant increased and decreased expression were identified. Heatmap for common DEGs was plotted with the pheatmap package in R.

Gene set enrichment analysis (GSEA) to estimate activated and repressed biological pathways in the comparison groups was performed using the clusterProfiler v.4.10.0 package for R.

lncRNAs subcellular localization analysis. The web services Locate-R (Ahmad et al., 2020) and iLoc-LncRNA 2.0 (Su et al., 2018) were used to determine the subcellular localization of lncRNAs.

Validation on an independent dataset. An independent microarray dataset from the GEO database (GSE63514; 24 normal samples, 40 CIN3 samples, and 28 cancer samples) was used to validate the obtained results. Gene expression was obtained using GEO2R GUI available on the sample panel in GEO (all comparison groups were likened to those performed on the original dataset). Differential expression analysis was performed within GEO2R

## Results

**Fig. 1.**
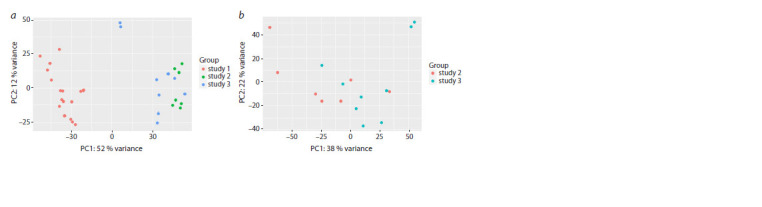
RNA sequencing data variability analysis using PCA before the exclusion of study No. 1 (a) and after its exclusion (b).

The variability analysis of RNA sequencing data and the PCA assessment of the batch effect presented in Figure 1 showed that study No. 1 was significantly different from studies No. 2 and No. 3 (Fig. 1a), which may indicate the presence of a batch effect. After it was excluded from the analysis, the variability of samples from different studies decreased significantly (Fig. 1b). In further analysis, only data from studies 2 and 3 were used. Samples from these two datasets were combined for analysis into a single dataset without normalization due to the overall low batch effect.

**Fig. 2.**
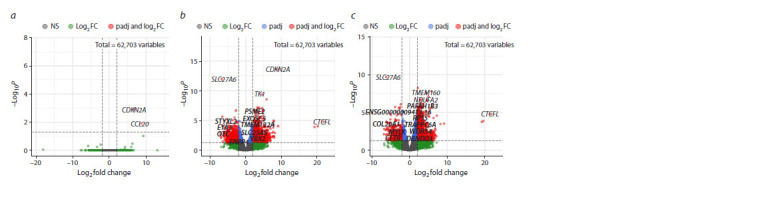
Volcano plots for differentially expressed genes in the “neoplasia vs. control” (a), “cancer vs. control” (b), and “cancer vs. neoplasia” (c) comparison groups.

The results of the differential gene expression analysis are shown in Figure 2. 1,572 DEGs were recorded in the “cancer vs. control” comparison, also 1,260 DEGs were recorded in the “cancer vs. neoplasia” comparison. It is important to note, that only 2 genes were observed to be differentially expressed in the “neoplasia vs. control” comparison

The genes with the largest difference in the expression level for all comparisons are shown in Table 2. The top 10 genes with increased and decreased expression for the “cancer vs. neoplasia” and “cancer vs. control” comparisons and 2 DEGs for the “neoplasia vs. control” comparison were presented. Out of the top 10 genes with increased expression for the “cancer vs. neoplasia” comparison, 1 belongs to the lncRNA class, 1 belongs to the piwi-interacting RNA (piRNA) class, 2 belong to the pseudogene class, 6 belong to the protein-coding gene class; out of the genes with decreased expression, 3 genes belong to the lncRNA class, 1 gene belongs to the piRNA class, and the remaining 6 belong to the protein-coding gene class. In turn, out of the top 10 genes with increased expression for the “cancer vs. control” comparison, 1 belongs to the piRNA class, and 9 belong to the protein-coding gene class; out of the genes with decreased expression, 4 belong to the lncRNA class, 1 belongs to the uncategorized transcript class, and the remaining 5 belong to the protein-coding gene class.

**Table 2.**
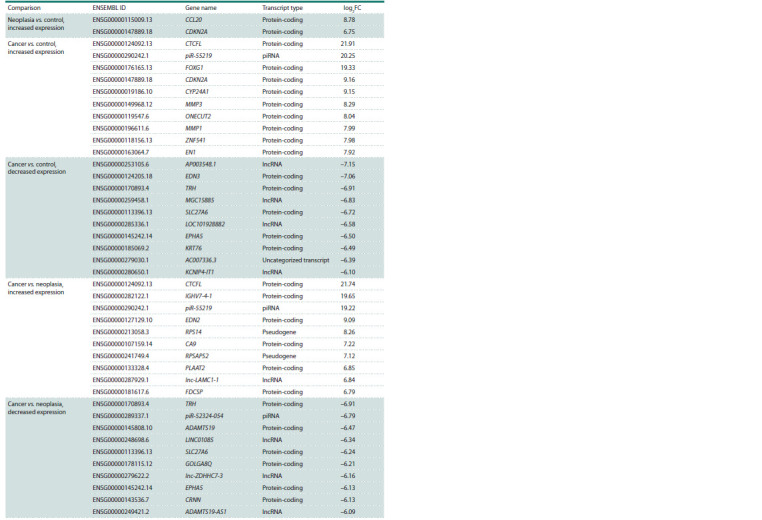
Top differentially expressed genes in the “neoplasia vs. control” , “cancer vs. control”, and “cancer vs. neoplasia” comparisons

**Fig. 3.**
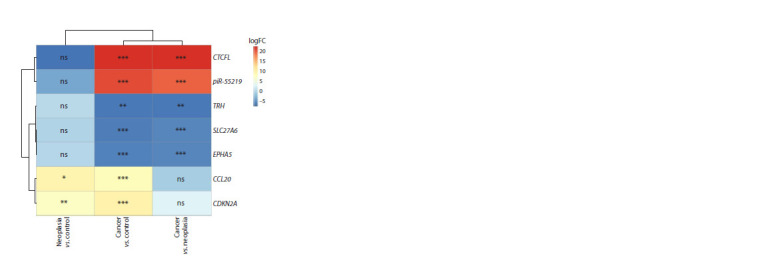
Heatmap of the expression change patterns by logFC of selected genes. The stars are intended to flag levels of significance for p-adjusted >0.05 (ns), <0.05 (*), <0.01 (**), <0.001 (***).

Search for common genes among the most strongly differentially expressed genes among all comparison groups resulted in the identification of an expression signature consisting of the genes CCL20, CDKN2A, CTCFL, piR-55219, TRH, SLC27A6 and EPHA5. The expression patterns of these genes are shown in Figure 3.

**Fig. 4.**
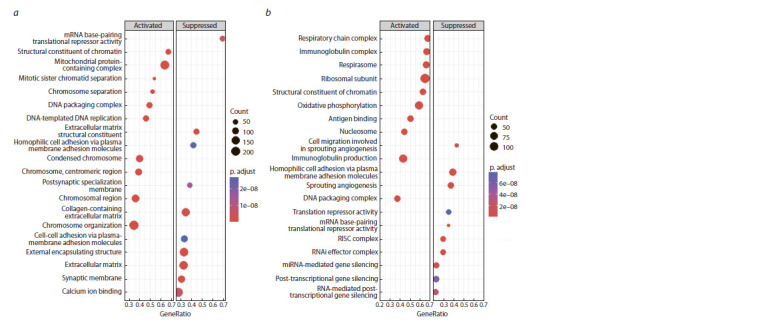
Results of gene set enrichment analysis for the “cancer vs. control” (a) and “cancer vs. neoplasia” (b) comparison groups

The gene enrichment analysis shown in Figure 4 demonstrated that in the “cancer vs. control” comparison, the molecular pathways associated with the cell cycle, DNA packaging, replication and translational mRNA base-pairing repression were activated, and the pathways associated with the membrane structure and cell-cell adhesion were repressed. Conversely, in the “cancer vs. neoplasia” comparison, molecular pathways related to the immunoglobulin production, antigen binding, respiratory chain and respirasome were activated, while pathways related to the translational mRNA base-pairing repression, post-transcriptional silencing, RISC and RNAi effector complexes were repressed

Analysis of the subcellular localization of differentially expressed lncRNAs using two different web resources (Table 3) showed that subcellular localization is identified ambiguously for ADAMTS19-AS1 (nucleus and exosome), which may be related to differences in the computational approaches by which Locate-R (Local Deep SVM approach) and iLoc-LncRNA 2.0 (SVM approach) are implemented, despite the fact that both models are based on an analysis of the RNALocate lncRNA localization database. The results clearly indicated cytoplasmic localization for most of the transcripts (Table 3).

**Table 3.**
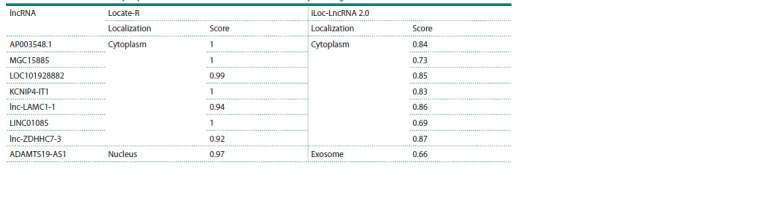
Results of differentially expressed lncRNAs subcellular localization analysis using Locate-R and iLoc-LncRNA 2.0

Validation on an independent dataset

Differential gene expression analysis on an independent dataset (Table 4) demonstrated that the number of DEGs observed in the “neoplasia vs. control” comparison was much higher than in the same comparison in our study, whereas for the other two comparisons, the number of DEGs was lower in the independent dataset than in our study. In particular, the CDKN2A and SLC27A6 genes confirmed their expression change in the same pattern after validation.

**Table 4.**
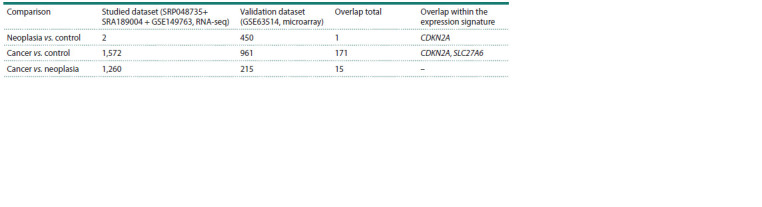
Comparative analysis of the DEGs in datasets for study and validation

## Discussion

The division of HPV-infected cervical epithelial cells leads to neoplastic tissue changes or cervical intraepithelial neoplasia (CIN). The changes detected at the levels of cell morphology and tissue structure are the consequence of alterations at the molecular level. Neoplastic changes of epithelial cells in HPV infection are characterized by an increased level of transcription of the CCL20 and CDKN2A genes, which lasts through the progression of the malignant process

Tumor development assumes long-term persistence of HPV and the formation of a high viral load. Moreover, the virus can use the replicative apparatus of human cells and avoid the action of immune system factors. The main mechanisms of evasion from the immune system include modulation of antigen presentation, inhibition of cytokines and chemoattractants, modulation of cell adhesion molecule synthesis and inhibition of antigen-presenting cell migration

The CCL20 gene, differentially expressed in neoplastic changes of infected cells, may be a direct participant in these processes. CCL20 belongs to the subfamily of small cytokine CC genes, it is located on chromosome 2q and contains 4 exons and 3 introns. This gene encodes macrophage inflammatory protein (MIP)-3α, predominantly expressed in liver, colon, prostate, cervix, and skin. It has been reported that endothelial cells, neutrophils, T helper 17 (Th17) cells, B cells, natural killer cells, dendritic cells (DC) and macrophages secrete CCL20 (Yamazaki et al., 2008; Nandi et al., 2014). CCL20 as a chemoattractant is involved in recruiting lymphocytes and dendritic cells to epithelial cells. It is believed that CCL20 may play an important role in the regulation of Langerhans cells, which are the main antigen-presenting cells for HPV presentation, causing an immune response.

From this perspective, it is reasonable to assume that active expression of CCL20 would be triggered in response to the appearance of human papillomavirus in the body. However, many studies indicate that HPV oncoproteins E6 and E7 may reduce the production of the chemokine CCL20 in keratinocytes by inhibiting its transcription. And thus, HPV, in an attempt to avoid an immune response, may negatively modulate the expression of this chemokine in the epithelium, thereby blocking the migration of inflammatory cells, such as Langerhans cells, to the lesion site (Guess, McCance, 2005; Wang et al., 2010; Jiang, Xue, 2015; Fernandes et al., 2021).

However, in the later stages of cervical carcinogenesis, the landscape changes and CIN3 lesions often contain myeloid cells such as macrophages and dendritic cells (Mazibrada et al., 2008) and, as a number of studies have shown, CCL20 levels in cervical cancer tissues are significantly higher than in non-tumor and normal control tissues (Yu et al., 2015). Cervical cancer cells have been found to instruct cervical fibroblasts to produce CCL20 (Walch-Ruckheim et al., 2015). The rationale is that although normal immune cells attack and suppress tumor cells, some immune cells that infiltrate cancer tissue lose their anti-tumor function and play a role in promoting tumor progression (Beatty, Gladney, 2015; Binnewies et al., 2018).

Alteration of the cell cycle of infected epitheliocytes is possible due to changes in the transcription level of the CDKN2A gene. CDKN2A is a cyclin-dependent kinase 2a inhibitor gene that, through the use of alternative reading frames, produces two major proteins: p16 (INK4), an inhibitor of cyclindependent kinase 2 which arrests the G1-S transition in the cell cycle, and p14 (ARF), which binds the p53-stabilizing protein MDM2 (Robertson, Jones, 1999). It is important to note that CDKN2A is overexpressed in various cancers, and often its expression level correlates with the number of mutations, microsatellite instability in the tumor genome, and immune infiltration in the tumor microenvironment (Chen Z. et al., 2021). However, a study of CDKN2A expression in cervical cancer cell lines performed by real-time PCR and western blotting showed that it was reduced; moreover, the authors concluded that CDKN2A inhibits cell proliferation and invasion in cervical cancer through the AKT-mTOR lactate dehydrogenase mediated pathway (Luan et al., 2021). Several bioinformatics studies analyzing RNA sequencing data of cer-vical cancer samples have found that CDKN2A is a kind of “nodal gene” of tumorigenesis through interactions with various transcription factors, signaling molecules and microRNAs (e. g. miR-424-5p and miR-9-5p) and is overexpressed in cervical carcinoma in the TCGA project (Zhao et al., 2018; Chen Z. et al., 2021). In our study, CDKN2A expression was upregulated in patients with both HPV-associated neoplasia and HPV-associated cervical cancer, which draws attention to the importance of a more thorough study of the expression pattern of this gene and the features of the above pathologic conditions.

At the same time, the pattern of CDKN2A methylation in cervical cancer is relatively well known; several meta-analyses have shown that CDKN2A hypermethylation (relative to control samples) can be an indicator of early disease progression (Li J. et al., 2016). CDKN2A methylation was found to gradually increase with disease progression from stage 1 neoplasia to cervical cancer (Wijetunga et al., 2016), which can also be used as a comparative marker of disease severity. We would like to emphasize the need for a study linking the expression and methylation status of CDKN2A in HPV-associated neoplasia and cervical cancer to expand the understanding of the functional role of CDKN2A regulation in these conditions.

The most significant reduction of expression level in cancer cells relative to both control and neoplasia was found for five transcripts: SLC27A6, EPHA5, TRH, CTCFL, and piR-55219.

The SLC27A6 gene encodes a fatty acid transfer protein through the cell membrane. Long-chain fatty acids are essential for various physiological processes. The function of SLC27A6 in cervical cancer has not, to our knowledge, been clarified. However, it is reported that SLC27A6 expression was decreased in esophageal squamous cell carcinoma and breast cancer cells as well as nasopharyngeal carcinoma cells compared to normal cells (Xu C.Q. et al., 2015; Yen et al., 2019). It was also observed that the methylation ratio of the SLC27A6 promoter was higher in nasopharyngeal carcinoma than in nonmalignant tissues (Xu C.Q. et al., 2015). On the contrary, SLC27A6 gene expression was increased in papillary thyroid carcinoma (Dai et al., 2020).

The function of the ephrin A5 receptor encoded by the EPHA5 gene in cervical cancer is also unclear. However, suppression of EPHA5 expression by methylation has been established for breast cancer (Fu et al., 2010), prostate cancer (Li S. et al., 2015), and colorectal cancer (Kober et al., 2011). The loss of EPHA5 expression was associated with the degree of serous ovarian carcinoma – the expression of this gene in cancer was reduced by 45 % in relation to neoplasia (Chen X. et al., 2016).

The TRH gene encodes a member of the thyrotropin-releasing hormone family involved in the hypothalamus–pituitary– thyroid axis which exhibits feedback of thyroid hormone, thereby regulating metabolic and immunological homeostasis. TRH has been well investigated in the type of cancer such as acute myeloid leukemia, and a correlation between risk groups and TRH expression was found, and it was discovered that patients with higher TRH expression were more sensitive to chemotherapy (Gao et al., 2022). Regarding CIN and cervical cancer, site-specific assessment of TRH gene methylation (cg01009664) was investigated for the detection of CIN2+ and demonstrated high sensitivity and specificity with clinician-collected samples, but not with the self-collected ones (Chaiwongkot et al., 2023). A similar analysis was also performed using screening of TRH cg01009664 methylation for prediction of oral squamous cell carcinoma and oropharyngeal squamous cell carcinoma (Puttipanyalears et al., 2018).

Conversely, the CTCFL and piR-55219 genes are significantly upregulated in their expression in both “cancer vs. neoplasia” and “cancer vs. control” comparisons.

The CTCFL gene, which is sometimes also called BORIS, is a paralog of the widely known CTCF transcription factor and is normally expressed in pre-meiotic male germ cells together with ubiquitously expressed CTCF being involved in the regulation of the testis-specific genes (Soltanian, Dehghani, 2018; Debaugny, Skok, 2020). Unlike CTCF, CTCFL is more frequently amplified or transcriptionally activated, rather than mutated in cancers, and in cervical cancer the aberrant expression of CTCFL is linked with the re-initiating promoter hypomethylation of this gene (Debaugny, Skok, 2020). Moreover, a study performed on the cervical cancer stem-like cells (CSCs)/cancer-initiating cells (CICs) claimed that BORIS sf6 (isoform from subfamily 6) is specifically expressed in cervical CSCs/CICs and has a role in the maintenance of CSCs/ CICs and proposed a peptide isoform BORIS C34_24(9) as a promising candidate for cervical CSC/CIC-targeting immunotherapy (Asano et al., 2016). Clinically, in cases of epithelial ovarian cancer and cervical cancer, high levels of BORIS expression were associated with poorer prognosis/less median survival times of patients and advanced cancer stages (Soltanian, Dehghani, 2018).

Way less is known about piwi-interacting RNA piR-55219. In general, piwi-interacting RNAs (piRNAs), which are

25–31 nucleotides in length, have been found to cluster at transposon loci in the genome and are thought to be critical for silencing these mobile genetic elements, via DNA methylation, to maintain genomic integrity in germline stem cells. Although they have only recently been identified in cancers, it is possible that the piRNAs that mediate transposon silencing during normal germline differentiation are hijacked in cancer cells to silence other parts of the genome, resulting in a tumorigenic state (Siddiqi, Matushansky, 2012; Suzuki et al., 2012). Unfortunately, no specific information on the involvement of piR-55219 in cancer processes has been shown, which emphasizes the need for a more detailed investigation to establish a functional relationship between piRNAs and other cancer-specific genes.

## Conclusion

We identified a predominantly cytoplasmic localization for the majority of differentially expressed lncRNAs. These lncRNAs can be involved in post-transcriptional regulation through their influence on the stability of mRNAs, act as translation regulators while forming mRNA-lncRNA complexes and can release miRNAs from their target genes as miRNA “sponges” (Xu Y. et al., 2023). All these processes may be impaired in cervical cancer, so it is important to further investigate the molecular mechanisms of function of lncRNAs selected in this study.

The results of our study differ significantly between the discovery and validation cohorts, which may be related to sample preparation protocols (FF+FFPE vs. cryosectioning) and expression assessment method (RNA-seq vs. microarray), which once again confirms the need to generate large protocol-uniformed datasets for studying neoplasia and cervical cancer at the same time.

Therefore, the analysis of differential gene expression in HPV-infected neoplasia and cervical cancer revealed a pattern of 7 genes with altered transcription levels. The increased transcription level of the CCL20 and CDKN2A genes occurs at the stage of neoplastic epithelial changes and persists in cervical cancer. The CDKN2A and SLC27A6 genes confirmed their expression change in the same patterns after validation on the independent microarray dataset.

## Conflict of interest

The authors declare no conflict of interest.
